# Simple and High-Throughput Quantification of Mono- and Bivalent Foot-and-Mouth Disease Virus Vaccine Antigens by Differential Scanning Fluorimetry

**DOI:** 10.3390/vaccines13070721

**Published:** 2025-07-02

**Authors:** Yanli Yang, Xiaojie Chen, Ming Li, Fei Xin, Yi Zhao, Chengfeng Zhang, Yiping Pan, Chuanyu He, Sun He

**Affiliations:** TECON Biopharmaceutical Co., Ltd., Urumqi 830010, Chinazhaoyi@tecon-bio.com (Y.Z.); panyiping@tecon-bio.com (Y.P.);

**Keywords:** differential scanning fluorimetry, FMDV, quantification, serotyping, quality control

## Abstract

Background/Objectives: An accurate quantification of the effective antigens from different serotypes is essential for the quality control of multivalent vaccines, but it remains challenging. Herein, we developed a simple and high-throughput method using differential scanning fluorimetry (DSF) for quantifying foot-and-mouth disease virus (FMDV) antigens in monovalent and bivalent vaccines. Methods: Purified serotypes A and O FMDV were used to establish and validate the method. The DSF parameters, including the dye concentration, thermal scanning velocity, and PCR tube material, were optimized at different FMDV concentrations. The established DSF method was validated for the quantification of monovalent and A/O bivalent FMDV, and was compared with the ultracentrifugation of 86 samples from different processing stages and serotypes. Results: The DSF showed that the melting temperature (*T*_m_) of type A (56.2 °C) was significantly higher than that of type O FMDV (50.5 °C), indicating that their *T*_m_ can be distinguished in bivalent antigens. After optimizing the DSF parameters, a strong correlation (*R*^2^ > 0.998) was observed between the 146S concentration and the maximum of the first derivative of the DSF fluorescence (d(RFU)/dT) for both serotypes A and O FMDV. The method demonstrated good reproducibility (RSD < 10%) and high sensitivity (limit of detection: 0.7 μg/mL). Using a multiple linear regression analysis, the simultaneous quantification of A and O FMDV in the bivalent mixtures achieved recovery rates of 82.4–105.5%, with an RSD < 10% for most of the samples. Additionally, the DSF results correlated well with the ultracentrifugation data (Pearson *ρ* = 0.9789), validating its accuracy and broad applicability. Conclusions: In summary, DSF represents a simple, rapid, and high-throughput tool for the quality control of monovalent and bivalent FMDV vaccines.

## 1. Introduction

Many infectious diseases are caused by different serotypes of pathogens, with little or no cross-protection between these serotypes. To address this, bivalent or multivalent vaccines combine antigens from several serotypes in the desired amounts and proportions to achieve broad protection [1]. Examples include influenza [2], human papillomavirus [3], foot-and-mouth disease virus (FMDV) [4,5], and, recently, COVID-19 vaccines [6]. However, quantifying serotype-specific antigens during vaccine production and storage remains a major challenge for ensuring potency and regulatory compliance.

Inactivated FMDV vaccines have effectively reduced substantial economic losses caused by FMDV in susceptible cloven-hoofed animals in the last two decades [7]. FMDV has seven serotypes (A, O, C, Asia 1, SAT 1, SAT 2, and SAT 3), with A, O, and Asia 1 being the most predominant and lacking cross-protection [8]. The intact virus, also known as the 146S particle, is critical for the potency of FMDV vaccines, but is prone to dissociating into non-protective 12S subunits [9,10]. As multivalent FMDV vaccines are increasingly being used, an accurate and simple method to quantify the viral antigens of different serotypes simultaneously is highly needed, since the total antigen content alone cannot efficiently guide quality control due to diverse viral stability.

Ultracentrifugation is regarded as the ‘gold standard’ to separate and analyze FMDV [11,12], yet its practical limitations include time-intensive protocols, operational complexity, and poor reproducibility. An enzyme-linked immunosorbent assay (ELISA) is highly sensitive and specific [13,14], but struggles to differentiate intact viral antigens from disassembled subunits or aggregates. High-performance size-exclusion chromatography (HPSEC) enables the rapid quantification of intact and degraded FMDV during purification and storage processes [15,16]; however, like ultracentrifugation, it fails to distinguish between different serotypes with similar diameters. Recent advances in capillary zone electrophoresis (CZE) have demonstrated its utility for quantifying antigens in monovalent and A/O bivalent FMDV vaccines [17]. Nevertheless, the expensive equipment and high requirements for capillary properties have limited its widespread application. Furthermore, both HPSEC and CZE require about 30 min per sample, which is rapid but insufficient for high-throughput determination in vaccine preclinical studies.

Differential scanning fluorimetry (DSF), a cost-effective and user-friendly technique, has gained prominence in protein thermostability studies [18,19]. DSF monitors the fluorescence changes in environmentally sensitive probes during protein denaturation [20]. It has enhanced sensitivity compared to differential scanning calorimetry, requires small sample volumes, and detects in a high-throughput way [21]. Several studies have been conducted to determine the melting temperature (*T*_m_) of FMDV by DSF [22,23]. Nevertheless, its application in antigen quantification remains underexplored, with limited reports on recombinant protein quantification in bacterial lysates [24]. Crucially, DSF requires only fluorescent dyes and standard instrumentation (e.g., real-time polymerase chain reaction systems), significantly lowering the technical barriers.

Taking FMDV as an example, we demonstrate the application of DSF for the simultaneous quantification of multiple serotypes and for different processing steps. Using serotype A and O FMDV as models, we establish and validate that the DSF-based platform is capable of accurately analyzing mono-/bivalent vaccine samples. The parallel validation with ultracentrifugation confirms that DSF achieves comparable accuracy while dramatically improving the operational efficiency. This approach provides a rapid and simple tool for antigen quantification in FMDV vaccine development pipelines.

## 2. Materials and Methods

### 2.1. Materials

FMDV A/AKT-III strain and O/Mya98/XJ/2010 strain, which were isolated in China, were cultivated in BHK-21 cells and inactivated by binary ethyleneimine, followed by removing cell debris by centrifugation. The inactivated cell culture crude was further subjected to several purification steps before the final formulation. SYBR Green II was obtained from Solarbio Biological Technology Co., Ltd. (Beijing, China). Benzonase was purchased from Sigma-Aldrich (St Louis, MO, USA). All other chemicals were analytical grade reagents, and solutions were prepared using Milli-Q water (Millipore, Bedford, MA, USA).

### 2.2. Purification and Characterization of Pure FMDV

#### 2.2.1. Purification of FMDV

Pure FMDV of serotypes A and O was prepared from inactivated cell culture crude by hydrophobic interaction chromatography and size-exclusion chromatography, as described elsewhere [25]. The viral purity was confirmed by 12% SDS-PAGE, and the particle structures were verified by transmission electron microscope (TEM) and HPSEC. The concentration of 146S was determined by the hitherto standard ultracentrifugation method [11,12].

#### 2.2.2. TEM and HPSEC Analyses

TEM used a Philips FEI Tecnai 20 TEM (Royal Philips Electronics, Amsterdam) after the samples were applied to a 400-mesh copper grid and negatively stained with 1% (*v*/*v*) uranyl acetate.

HPSEC analysis was conducted using a TSK G4000 SW_XL_ (300 mm × 7.8 mm I.D.) analytical column (Tosohaas, Stuttgart, Germany) connected to an Agilent 1260 Infinity LC system (Agilent Technologies, Santa Clara, CA, USA). For each measurement, 100 μL of the sample was injected and eluted at 0.6 mL/min. The mobile phase was 50 mM phosphate buffer (pH 7.0) containing 100 mM Na_2_SO_4_ [22].

#### 2.2.3. *T*_m_ Determination by DSF

The *T*_m_ values of serotype O and A FMDV were measured to investigate the feasibility of using DSF for differentiating between the two viruses. The purified FMDV with known 146S concentration was mixed with SYBR Green II to a 2000-fold dilution of the commercial stock solution. A 40 μL mix was loaded onto a MicroAmp^®^ Fast 96-well reaction plate (Thermo Fisher Scientific, Waltham, MA, USA) and capped with optical cap strips (Bio-Rad, Hercules, CA, USA) to prevent sample evaporation. The plate was heated from 25 °C to 85 °C at a rate of 0.3 °C/3 s, and fluorescence was recorded at 0.3 °C using a BioRad CFX96 thermal cycler (Bio-Rad, Hercules, CA, USA) in SYBR mode. *T*_m_ of 146S was obtained according to the maximum of the first derivative (d(RFU)/dT) of the fluorescence curve. The background buffer was used as the control. To further confirm the *T*_m_ signal attribution, detection was stopped at 65 °C, and the mixture was taken out for further HPSEC analysis.

### 2.3. Optimization of DSF Parameters

Purified serotype O FMDV with 146S concentrations of 11–110 μg/mL was used for parameter optimization. Key variables, including dye concentration, thermal scanning velocity, and PCR tube material, were evaluated to maximize DSF signal quality. Dye dilution was 200-, 500-, 1000-, and 2000-fold of each commercial stock solution. Three heating protocols were compared: 0.3 °C/2 s ramp with fluorescence acquisition every 0.3 °C (method 3-2), 0.2 °C/3 s ramp with fluorescence acquisition every 0.2 °C (method 2-3), and 0.3 °C/3 s ramp with fluorescence acquisition every 0.3 °C (method 3-3). Three kinds of PCR tubes were assessed, including MicroAmp^®^ Fast 96-well reaction plate ((Thermo Fisher Scientific, Waltham, MA, USA), clear PCR tube (Eppendorf, Hamburg, Germany), and white low-profile PCR tube (Bio-Rad, Hercules, CA, USA).

### 2.4. DSF Method Validation

#### 2.4.1. Monovalent FMDV Quantification

Eight concentration levels were determined by DSF in the range of 0.7–89 μg/mL for serotype A and 0.7–110 μg/mL for serotype O FMDV. Calibration curves between the 146S concentration and the maximum d(RFU)/dT were obtained to evaluate the quantitative linearity. Precision was validated by three replicates randomly positioned within the instrument at each concentration level. The lower limit of detection (LOD) of DSF was determined according to a signal-to-noise ratio of 3.

#### 2.4.2. Fitting and Quantification of A and O FMDV Mixtures

To verify the applicability of DSF to quantify bivalent FMDV, purified serotype A (84.8 µg/mL) and O FMDV (105.7 µg/mL) were vortex-mixed with a volume ratio of 6:1, 5:2, 4:3, 3:4, 2:5, and 1:6. The mixtures were then analyzed by DSF under optimum conditions. The first-derivative plot for each serotype of FMDV was obtained by multiple linear regression analysis as follows:

(1) *Y* = *β*_1_ × *X*_A_ + *β*_2_ × *X*_O_

where *Y* represents the first-derivative plot of bivalent FMDV, and *X*_A_ and *X*_O_ represent the first-derivative plots of the purified serotype A and O FMDV, respectively. The values of *β*_1_ and *β*_2_ were fitted by Origin 8.0 software. The maximum values of the fitted first derivative for each serotype of FMDV were then compared to the calibration curves described in Section 2.4.1 to calculate the 146S concentration.

### 2.5. Application to Processing of Samples and Concordance Analysis with Ultracentrifugation

To assess the applicability of DSF for FMDV quantification in different processing steps, 86 samples from cell culture crude, inactivated crude, semi-purified, and purified FMDV of serotype A or O were detected by DSF. Additional benzonase digestion was performed for unpurified samples to eliminate interference from residual nucleic acid impurities [18]. Samples were treated with 200 U/mL benzonase and incubated at room temperature for 10 min before DSF detection. The results were compared to the ultracentrifugation method. Concordance correlation coefficient was analyzed using MedCalc version 11.6 (MedCalc Software, Ostend, Belgium).

## 3. Results

### 3.1. Preparation and Characterization of Pure FMDV

**Figure 1:**
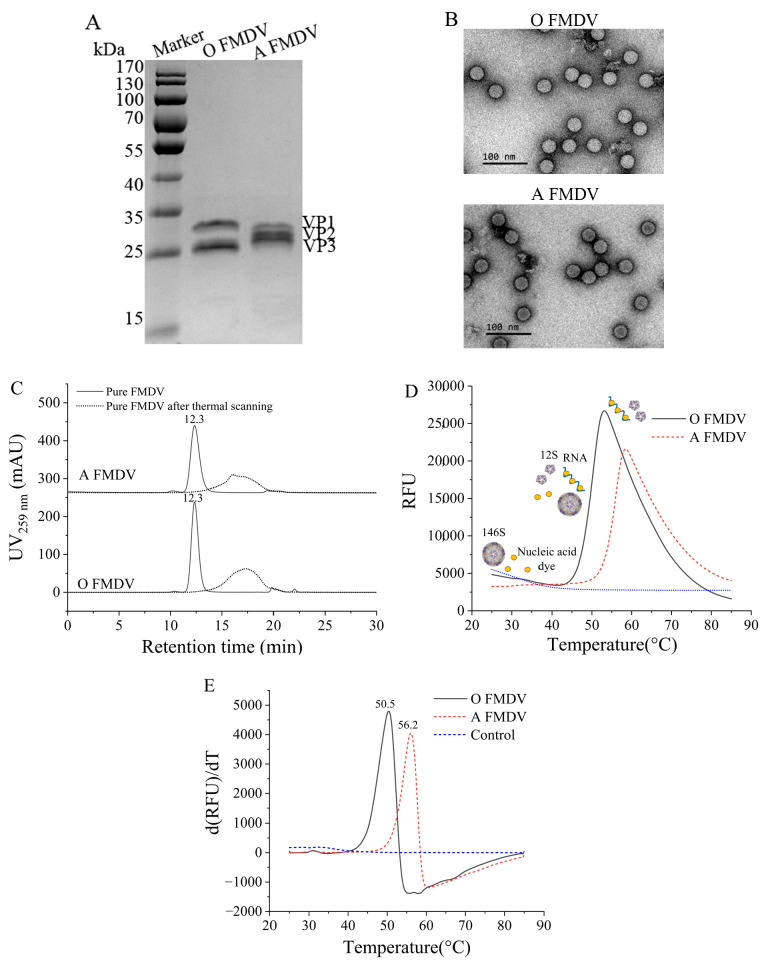
Characterization of the purified serotype A and O FMDV. The purity and particle structures were identified by (**A**) SDS-PAGE, (**B**) TEM, and (**C**) HPSEC. (**D**) The DSF signals and schematic illustration of the RNA release and binding with the florescent dye to trigger a strong fluorescence signal upon the thermal-induced dissociation of 146S. (**E**) The first-derivative plots of the fluorescence curves of the FMDV. The FMDV after DSF scanning up to 65 °C was reanalyzed by (**C**) HPSEC.

The SDS-PAGE analysis confirmed the high purity of both serotype A and O FMDV (Figure 1A). Only three protein bands, corresponding to the structural proteins VP1, VP2, and VP3, were observed for each serotype. The TEM analysis revealed both serotypes shared similar particle sizes of 28 nm (Figure 1B), consistent with previous reports [22], and the HPSEC (Figure 1C) also showed identical retention times of 12.3 min [26]. Figure 1D shows the fluorescence curves used in the DSF determination and an illustration of the detection process. Prior to thermal denaturation (25–50 °C), the compact 146S structure prevented the SYBR Green II from binding to the viral RNA, resulting in minimal fluorescence. As the temperature increased up to a certain point, the 146S began to dissociate into 12S and release RNA, which bound to the dyes and generated a fluorescence surge. The fluorescence intensity was expected to be positively correlated with the viral RNA content. Then, the fluorescence signal plateaued upon complete virus dissociation and gradually quenched at higher temperatures. The control, containing the background buffer and dye, showed no evident DSF signals (Figure 1C,D), verifying that the DSF signals were derived from the FMDV. The HPSEC analysis after DSF scanning up to 65 °C confirmed the 146S dissociation, as evidenced by the disappearance of the 12.3 min peak and the emergence of 12S/RNA complexes (Figure 1C) [22]. The *T*_m_ was defined as the temperature corresponding to the maximum d(RFU)/dT of the fluorescence curve. As shown in Figure 1E, the *T*_m_ of type A (56.2 °C) was significantly higher than that of type O FMDV (50.5 °C), suggesting that their *T*_m_ can be distinguished in bivalent antigens. Despite their structural similarities, serotype A exhibited a significantly higher thermal stability than serotype O, likely due to the divergent amino acid sequences in their structural proteins [27].

### 3.2. Development of DSF Method

**Figure 2:**
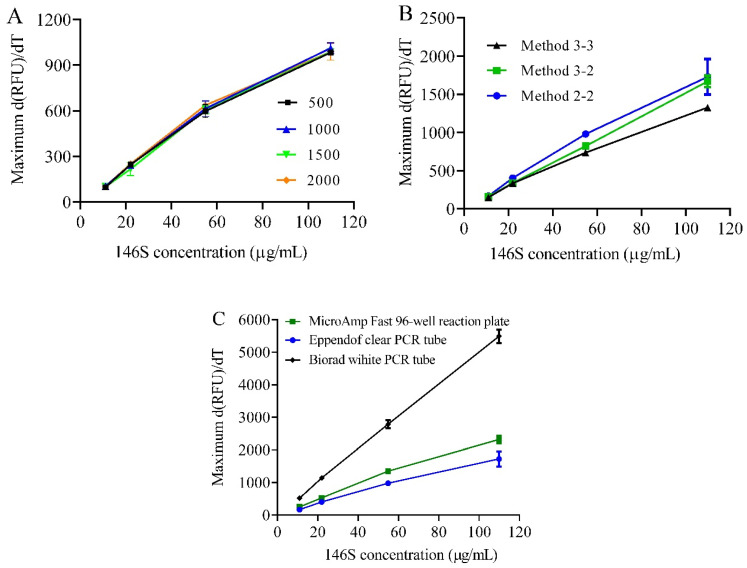
DSF method development with purified serotype O FMDV. The optimized conditions include the (**A**) dilution fold of SYBR Green II, (**B**) thermal scanning velocity, and (**C**) material of the PCR tube. The effects of these conditions on the maximum d(RFU)/dT are evaluated.

To optimize the DSF method for detecting low concentrations of 146S in inactivated FMDV vaccines, typically <100 µg/mL, various conditions were evaluated using pure serotype O FMDV. The maximum d(RFU)/dT under different dilution folds of SYBR Green II (Figure 2A), thermal scanning rates (Figure 2B), and PCR tube materials (Figure 2C) were compared across a range of 146S concentrations. Excess dye relative to the viral RNA content was ensured by testing 200- to 2000-fold dilutions. A 2000-fold dilution achieved consistent maximum d(RFU)/dT values for concentrations up to 110 µg/mL, confirming sufficient dye. The maximum d(RFU)/dT showed a positive correlation with the 146S concentration under all the detected dye dilution folds, further verifying the signals were derived from the 146S. To balance precision and efficiency, we set the detector to record the fluorescence every 0.2 or 0.3 °C increase in temperature, with a heating rate of (0.2–0.3) °C/(2–3) s. The total analysis times from 25 °C to 85 °C were 1 h 40 min for method 2-2, 1 h 10 min for method 3-2, and 1 h for method 3-3, corresponding to heating rates of 37.5 °C/h–60 °C/h. Although method 2-2 yielded the highest maximum d(RFU)/dT, it was time-consuming. Method 3-2 was selected as the optimal method due to its balance of efficiency and sensitivity. The assessment of three widely used PCR tubes showed that the white tubes yielded significantly higher signals than the clear tubes, likely due to better fluorescence reflection. Among the parameters studied, the material of the PCR tubes had the greatest impact on the results.

The final optimized conditions were a 1000-fold dilution of the commercial SYBR Green II stock solution, heating from 25 °C to 85 °C at a rate of 0.3 °C/3 s with fluorescence acquisition every 0.3 °C, and using white PCR tubes.

### 3.3. Method Validation for Quantification of Monovalent and Bivalent FMDV

**Figure 3:**
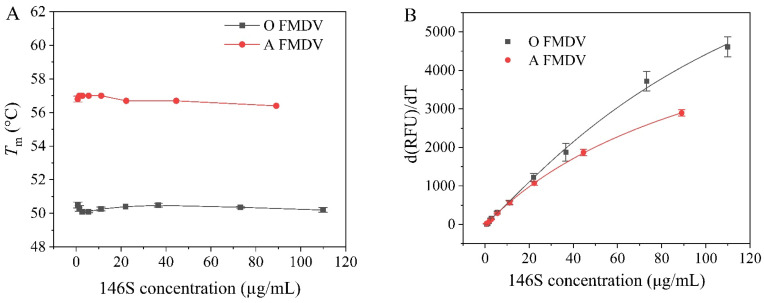
DSF analysis of the purified serotypes A and O at different 146S concentrations. (**A**) The determined *T*_m_ values. (**B**) The calibration curves between the 146S concentration and the maximum d(RFU)/dT. The 146S concentration was 0.7–89 μg/mL and 0.7–110 μg/mL for serotypes A and O, respectively. Three replicates at each concentration level were randomly placed at different positions. All the analyses were performed at the optimized DSF conditions.

Pure A and O FMDV of known concentrations predetermined by HPSEC were serially diluted and detected by DSF under the optimal conditions to build a calibration curve and determine the LOD for each serotype. As shown in Figure 3A, the *T*_m_ values demonstrated minor concentration-dependent variations, consistent with previous reports on the concentration effects in thermal stability assays [28]. Nevertheless, these variations did not significantly alter the characteristic *T*_m_ differences between the two serotypes. The maximum d(RFU)/dT values increased with an increasing concentration of 146S for both serotypes, but not in a linear manner (Figure 3B). A four-parameter logistic regression revealed strong correlations between the 146S concentrations and the maximum d(RFU)/dT values for both serotype A (*R*^2^ = 0.999) and O FMDV (*R*^2^ = 0.998). The method demonstrated satisfactory reproducibility, with inter-assay relative standard deviation (RSD) values below 10% and a sensitivity with LOD of 0.7 μg/mL for both serotypes.

**Figure 4:**
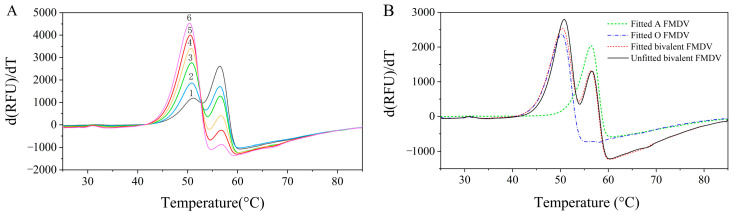
DSF analysis of pure serotype A and O FMDV mixtures at different concentrations. (**A**) The first-derivative plots of fluorescence curves 1–6: purified serotype A and O FMDV samples with 146S concentrations of 84.8 µg/mL and 105.7 µg/mL, respectively, were vortex-mixed with volume ratios of 6:1, 5:2, 4:3, 3:4, 2:5, and 1:6. (**B**) The fitted first-derivative plots of group 4 by multiple linear regression analysis.

The analytical performance was further validated on bivalent samples containing predetermined serotype A and O FMDV ratios. While the first-derivative plots showed distinct *T*_m_ values for each serotype (Figure 4A), the overlapping thermal transitions made it difficult to determine the individual d(RFU)/dT maxima accurately. The d(RFU)/dT of serotype A FMDV became negative, particularly when it was less prevalent. A multiple linear regression analysis successfully resolved the overlapping signals. The reprehensive fitting plots for group 4 are shown in Figure 4B. The regression model well fitted the data, with an adjusted *R*^2^ of 0.9897 and an *F* value of 9678.16 (*p* < 0.05). The subsequent quantification was performed using serotype-specific calibration curves as obtained from Figure 2B, and the results are summarized in Table 1. The method demonstrated acceptable accuracy for bivalent vaccine analysis, with recovery rates of 82.4–105.5% for serotype A and 90.2–101.2% for serotype O, coupled with a precision below 10% RSD for most measurements. Notably, the precision for serotype A exceeded 10% RSD in Group 6, where its concentration was significantly lower. The sequential thermal denaturation profiles (serotype O preceding A) could cause signal interference through O-type fluorescence quenching during A-type detection, particularly at low A:O concentration ratios. This may explain the underestimation of serotype A content and the increased variability in Group 6. Nevertheless, as multivalent FMDV vaccines maintain approximately equivalent antigen levels across serotypes [29,], the performance remains acceptable for quality control. Significant deviations from the expected concentration ratios would inherently indicate product degradation, thereby reducing concerns about marginal quantification inaccuracies.

### 3.4. Application to Processing of Samples and Concordance Analysis with Ultracentrifugation

**Figure 5:**
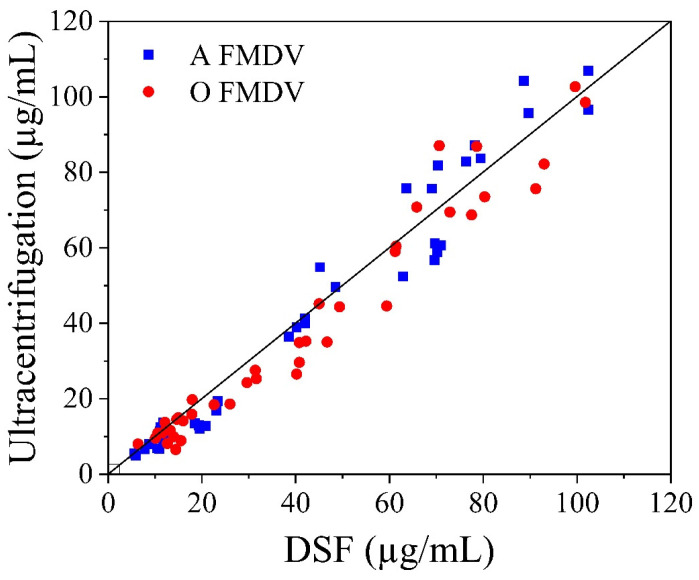
Concordance correlation plot for 146S quantification by DSF and ultracentrifugation. Each symbol represents results corresponding to samples from different processing steps containing either serotype A or O FMDV.

The applicability of DSF for quantifying FMDV in processing samples was assessed by 86 samples containing cell culture crude, inactivated crude, and semi-purified and purified samples of type O and A FMDV. The cell culture crude contained a large amount of host cell nucleic acids, which would result in high background signals obscuring the signals from the 146S. Therefore, benzonase digestion was necessary before DSF detection of the cell culture crude before and after inactivation. All four steps the FMDV samples went through could be detected with the *T*_m_ peaks (see Supplementary Materials), although a further baseline correction was needed. The results are compared with those from ultracentrifugation in Figure 5. Some deviations were observed between the two methods, possibly due to differences in sample purity across the processing steps and batches. However, the statistical analysis indicated that the results obtained by these two methods were well correlated, with a concordance correlation coefficient = 0.9761, Pearson *ρ* (precision) = 0.9789, and bias correction factor *C*_b_ (accuracy) = 0.9971. These results indicate that DSF can be applied for rapid and high-throughput quantification in FMDV processing.

## 4. Discussion

Despite the widespread application of DSF in protein thermostability studies, its utility for antigen quantification remains underexplored. This work expands DSF’s utility beyond traditional protein stability assessments, establishing it as a versatile platform for vaccine development.

FMDV quantification via DSF was accomplished through real-time fluorescence monitoring of the nucleic acid dyes bound to the thermally released viral RNA. The fluorescence intensity was expected to be positively correlated with the viral RNA content. While the Δ (fluorescence intensity) was used to quantify the protein concentrations in a previous study [24], we employed the first derivative of the DSF fluorescence and the maximum d(RFU)/dT to quantify FMDV. The maximum d(RFU)/dT was shown to be strongly correlated with the 146S concentration (*R*^2^ > 0.998) and also monitored the structural integrity (via the *T*_m_ shifts), thus providing dual quality indicators. More importantly, the close *T*_m_ values between serotype A and O FMDV led to overlapping fluorescence signals during thermal scanning. This spectral overlap significantly impeded the accurate discrimination of the individual fluorescence intensities, thereby compromising the precise quantification of each serotype. While the d(RFU)/dT demonstrated enhanced sensitivity and capability for resolving the characteristic melting signatures, the complete separation of these overlapping signals remained a challenge (Figure 4A). To address this, a multiple linear regression analysis using the first-derivative plots of the purified serotype A and O FMDV was established to obtain signals for each serotype of bivalent antigens. The recovery rates obtained for both serotypes confirmed the method’s feasibility.

Unlike ultracentrifugation and chromatographic methods that rely on physical analyte separation, DSF operates as an in situ technique leveraging nucleic acid-specific dye selectivity. Proteinaceous impurities and other non-nucleic acid contaminants demonstrate no dye interaction, thereby eliminating interference in FMDV detection. The reported LOD for the HPSEC quantification of FMDV was below 0.6 μg/mL (S/N of 37) [15]. No precise LOD for ultracentrifugation was available, but a sample with a low concentration of 1.85 μg/mL was detected by ultracentrifugation [12]. The LOD of 0.7 μg/mL for the DSF method indicates a comparable sensitivity to these two techniques. The quantification range of 0.7–110 μg/mL by DSF covers the concentration ranges across different production stages of most viral vaccine products. Despite the limitations, such as signal interference from highly impure samples, the advantages of DSF could make it a transformative tool for high-throughput screening during process development. Compared to ultracentrifugation and chromatography-based methods, the DSF approach offers distinct advantages:

• High-throughput capability: a single DSF run (~1 h) enables the simultaneous analysis of up to 96 samples, significantly surpassing the throughput of HPSEC (~30 min/sample) and ultracentrifugation (>4 h/sample).
• Cost-effectiveness: this method eliminates the requirements for specialized columns, antibodies, or ultracentrifugation equipment, reducing the operational costs compared to CZE and HPSEC.
• Serotype discrimination: Leveraging the intrinsic differences in the thermal stability between serotypes (e.g., Δ*T*_m_≈5.7 °C for A vs. O FMDV), DSF simultaneously quantifies multivalent antigens—a critical advancement over size-based separation methods. This allows for the more accurate evaluation of different serotype antigens in vaccines.
• Simplified workflow: minimal sample pretreatment (e.g., benzonase digestion for crude samples) and automated data acquisition make DSF accessible for routine quality control across vaccine production stages.

Future studies should explore DSF’s adaptation to adjuvanted and higher-valency FMDV vaccines, as well as other thermally distinguishable viruses (e.g., influenza, SARS-CoV-2 variants) and combination vaccines.

Adjuvanted vaccines were not included in this study due to the diversity of commercial product formulations, which comprise variations in the adjuvant types, FMDV serotypes, and buffer compositions. Notably, some adjuvants can interfere with DSF detection [22]. Oil emulsions (e.g., ISA206 VG) are widely used in FMDV vaccines [30]. An adjuvanted emulsion contains a large number of droplets, giving vaccines a milky-white appearance that causes a strong fluorescence reflection and interferes with detection. Additionally, adjuvants that interact strongly with dyes also disturb DSF detection. While DNA adjuvants, such as cytidine–phosphate–guanosine oligodeoxynucleotides (CpG ODN), have been reported to enhance FMDV vaccine efficacy [31], they generate high background fluorescence, obscuring FMDV signals. However, these interferences can be minimized by removing the adjuvants. For instance, antigens can be extracted from an emulsion vaccine by adding organic solvents like pentanol [17]. Similarly, DNA adjuvants can be removed by benzonase digestion or a specific adsorption step. Nonetheless, such treatments require rigorous validation to ensure antigen preservation.

Other FMDV serotypes have also been analyzed by DSF in thermal stability studies [32], including Asia 1 (53.2 °C), C (41.3 °C), SAT 1 (44 °C), SAT 2 (46.3 °C), and SAT 3 (48.5 °C). These serotypes showed similar DSF signals, except for the differences in the *T*_m_ values. Therefore, it is reasonable to speculate that they could also be quantified using the DSF method, once their calibration curves are provided. Serotypes that exhibit a sufficient Δ*T*_m_, such as Asia 1 vs. the other four serotypes, should be quantifiable simultaneously in bivalent vaccines. It should be noted that *T*_m_ variations occur across strains within the same serotype and are highly sensitive to buffer conditions (e.g., pH and ionic strength) [27]. Consequently, multivalent vaccine analysis requires consideration of both the strain-specific thermal stability and formulation parameters. Although higher-valency vaccines were not studied in this work, the quantifiability also depends on whether the strains exhibit distinguishable *T*_m_ signals. Integration with machine learning algorithms may help to enhance the multi-peak resolution of complex mixtures.

## 5. Conclusions

In conclusion, we established a simple DSF method with good accuracy and precision for the high-throughput quantification of 146S antigens in monovalent and bivalent FMDV samples. This method can be used as a platform for vaccine development and quality control of monovalent and bivalent vaccines.

## Figures and Tables

**Table 1:** Quantification of serotype A and O FMDV mixtures by DSF.

Group	Serotype	Theoretical Concentration (μg/mL)	DSF Quantification (*n* = 3)		
			Concentration (μg/mL)	RSD (%)	Recovery Rate (%)
1	A	72.7	74.7 ± 4.2	5.6	102.8
	O	15.1	13.7 ± 0.6	4.4	90.6
2	A	60.6	63.9 ± 3.3	5.2	105.5
	O	30.3	30.7 ± 0.8	2.5	101.2
3	A	48.4	45.6 ± 0.6	1.3	94.1
	O	45.4	42.2 ± 0.2	0.6	92.9
4	A	36.3	31.6 ± 0.9	2.9	87.3
	O	60.6	57.7 ± 0.6	1.1	95.2
5	A	24.2	19.9 ± 0.2	1.2	82.4
	O	75.7	73.9 ± 2.0	2.7	97.6
6	A	12.1	10.1 ± 1.3	13.1	83.6
	O	90.9	91.9 ± 0.9	9.8	101.1

## Data Availability

Data will be provided upon reasonable request.

## Supplementary Materials

The following supporting information can be downloaded at: https://www.mdpi.com/article/10.3390/vaccines13070721/s1, Figure S1: The first-derivative plots of the DSF fluorescence curves of FMDV from different processing steps.

## Abbreviations

The following abbreviations are used in this manuscript:

DSF	Differential scanning fluorimetry
FMDV	Foot-and-mouth disease virus
ELISA	Enzyme-linked immunosorbent assay
HPSEC	High-performance size-exclusion chromatography
CZE	Capillary zone electrophoresis
Tm	Melting temperature
LOD	Lower limit of detection
TEM	Transmission electron microscope
RSD	Relative standard deviation

## References

[1] Schlingmann B., Castiglia K.R., Stobart C.C., Moore M.L. (2018). Polyvalent vaccines: High-maintenance heroes. PLoS Pathog..

[2] Park J., Fong Legaspi S.L., Schwartzman L.M., Gygli S.M., Sheng Z.M., Freeman A.D., Matthews L.M., Xiao Y.L., Ramuta M.D., Batchenkova N.A. (2022). An inactivated multivalent influenza A virus vaccine is broadly protective in mice and ferrets. Sci. Transl. Med..

[3] Wang D.N., Liu X.L., Wei M.X., Qian C.Y., Song S., Chen J., Wang Z.P., Xu Q., Yang Y.R., He M.Z. (2020). Rational design of a multi-valent human papillomavirus vaccine by capsomere-hybrid co-assembly of virus-like particles. Nat. Commun..

[4] Peta F.R.M., Sirdar M.M., van Bavel P., Mutowembwa P.B., Visser N., Olowoyo J., Seheri M., Heath L. (2021). Evaluation of potency and duration of immunity elicited by a multivalent FMD vaccine for use in South Africa. Front. Vet. Sci..

[5] Childs K., Harvey Y., Waters R., Woma T., Wilsden G., Sun H., Sun P., Seago J. (2023). Development of a quadrivalent foot-and-mouth disease vaccine candidate for use in East Africa. Vaccine.

[6] Chang S., Shin K.S., Park B., Park S., Shin J., Park H., Jung I.K., Kim J.H., Bae S.E., Kim J.O. (2024). Strategy to develop broadly effective multivalent COVID-19 vaccines against emerging variants based on Ad5/35 platform. Proc. Natl. Acad. Sci. USA.

[7] Kamel M., El-Sayed A., Vazquez H.C. (2019). Foot-and-mouth disease vaccines: Recent updates and future perspectives. Arch. Virol..

[8] Diaz-San Segundo F., Medina G.N., Stenfeldt C., Arzt J., de los Santos T. (2017). Foot-and-mouth disease vaccines. Vet. Microbiol..

[9] Bachrach H.L., Moore D.M., McKercher P.D., Polatnick J. (1975). Immune and antibody responses to an isolated capsid protein of foot-and-mouth disease virus. J. Immunol..

[10] Harmsen M.M., Fijten H.P.D., Westra D.F., Dekker A. (2015). Stabilizing effects of excipients on dissociation of intact (146S) foot-and-mouth disease virions into 12S particles during storage as oil-emulsion vaccine. Vaccine.

[11] Barteling S.J., Meloen R.H. (1974). A simple method for the quantification of 140S particles of foot-and-mouth disease virus (FMDV). Arch. Gesamte Virusforsch..

[12] Kim A.-Y., Park S.Y., Park S.H., Kim J.Y., Jinm J.S., Kim E.-S., Park J.H., Ko Y.J. (2022). Comparison of high-performance liquid chromatography with sucrose density gradient ultracentrifugation for the quantification of foot-and-mouth disease vaccine antigens. Vaccines.

[13] Harmsen M.M., Fijten H.P., Westra D.F., Coco-Martin J.M. (2011). Effect of thiomersal on dissociation of intact (146S) foot-and-mouth disease virions into 12S particles as assessed by novel ELISAs specific for either 146S or 12S particles. Vaccine.

[14] Feng X., Ma J.-W., Sun S.-Q., Guo H.-C., Yang Y.-M., Jin Y., Zhou G.-Q., He J.-J., Guo J.-H., Qi S.-Y. (2016). Quantitative detection of the foot-and-mouth disease virus serotype O 146S antigen for vaccine production using a double-antibody sandwich ELISA and nonlinear standard curves. PLoS ONE.

[15] Spitteler M.A., Romo A., Magi N., Seo M.G., Yun S.J., Barroumeres F., Régulier E.G., Bellinzoni R. (2019). Validation of a high performance liquid chromatography method for quantitation of foot-and-mouth disease virus antigen in vaccines and vaccine manufacturing. Vaccine.

[16] Yang Y.L., Li H., Li Z.J., Zhang Y., Zhang S.P., Chen Y., Yu M.R., Ma G.H., Su Z.G. (2015). Size-exclusion HPLC provides a simple, rapid, and versatile alternative method for quality control of vaccines by characterizing the assembly of antigens. Vaccine.

[17] Song Y.M., Yang Y.L., Lin X., Zhao Q.Z., Li Z.J., Ma G.H., Su Z.G., Zhang S.P. (2021). On-line separation and quantification of virus antigens of different serotypes in multivalent vaccines by capillary zone electrophoresis: A case study for quality control of foot-and-mouth disease virus vaccines. J. Chromatogr. A.

[18] McClure S.M., Ahl P.L., Blue J.T. (2018). High throughput differential scanning fluorimetry (DSF) formulation screening with complementary dyes to assess protein unfolding and aggregation in presence of surfactants. Pharm. Res..

[19] Gooran N., Kopra K. (2024). Fluorescence-based protein stability monitoring-A Review. Int. J. Mol. Sci..

[20] Bruce D., Cardew E., Freitag-Pohl S., Pohl E. (2019). How to stabilize protein: Stability screens for thermal shift assays and nano differential scanning fluorimetry in the virus-X project. Jove-J. Vis. Exp..

[21] Senisterra G.A., Finerty P.J. (2009). High throughput methods of assessing protein stability and aggregation. Mol. Biosyst..

[22] Song Y.M., Yang Y.L., Lin X., Li X.N., Zhang X., Ma G.H., Su Z.G., Zhang S.P. (2020). In-situ and sensitive stability study of emulsion and aluminum adjuvanted inactivated foot-and-mouth disease virus vaccine by differential scanning fluorimetry analysis. Vaccine.

[23] Li H., Liu P., Dong H., Dekker A., Harmsen M.M., Guo H., Wang X., Sun S. (2024). Foot-and-mouth disease virus antigenic landscape and reduced immunogenicity elucidated in atomic detail. Nat. Commun..

[24] Seo D.-H., Jung J.-H., Kim H.-Y., Park C.-S. (2014). Direct and simple detection of recombinant proteins from cell lysates using differential scanning fluorimetry. Anal. Biochem..

[25] Li H., Yang Y.L., Zhang Y., Zhang S.P., Zhao Q., Zhu Y.Y., Zou X.Q., Yu M.R., Ma G.H., Su Z.G. (2015). A hydrophobic interaction chromatography strategy for purification of inactivated foot-and-mouth disease virus. Protein Expres Purif..

[26] Song Y.M., Yang Y.L., Lin X., Zhao Q.Z., Su Z.G., Ma G.H., Zhang S.P. (2022). Size exclusion chromatography using large pore size media induces adverse conformational changes of inactivated foot-and-mouth disease virus particles. J. Chromatogr. A.

[27] Jin J.S., Lee G., Kim J.Y., Lee S., Park J.-H., Park S.Y., Ko Y.J. (2024). Calcium Chloride as a novel stabilizer for foot-and-mouth disease virus and its application in the vaccine formulation. Vaccines.

[28] Wen J., Arthur K., Chemmalil L., Muzammil S., Gabrielson J., Jiang Y. (2012). Applicationsof differential scanning calorimetry for thermal stability analysis of proteins:qualification of DSC. J. Pharm. Sci.

[29] Kim D.-W., Cho G., Kim H., Lee G., Lim T.-G., Kwak H.-Y., Park J.-H., Park S.-H. (2023). Immunogenicity and protection against foot-and-mouth disease virus in swine intradermally vaccinated with a bivalent vaccine of foot-and-mouth disease virus type O and A. Vaccines.

[30] Charerntantanakul W. (2020). Adjuvants for swine vaccines: Mechanisms of actions and adjuvant effects. Vaccine.

[31] Ren J., Yang L., Xu H., Zhang Y., Wan M., Liu G., Zhao L., Wang L., Yu Y. (2011). CpG oligodeoxynucleotide and montanide ISA 206 adjuvant combination augments the immune responses of a recombinant FMDV vaccine in cattle. Vaccine.

[32] Kotecha A., Zhang F., Juleff N., Jackson T., Perez E., Stuart D., Fry E., Charleston B., Seago J. (2016). Application of the thermofluor PaSTRy technique for improving foot-and-mouth disease virus vaccine formulation. J. Gen. Virol..

