# Supplementary material for: Simple and High-Throughput Quantification of Mono- and Bivalent Foot-and-Mouth Disease Virus Vaccine Antigens by Differential Scanning Fluorimetry
Source: Vaccines (Basel). 2025 Jul 2;13(7):721. doi: 10.3390/vaccines13070721 (PMC12298750; doi:10.3390/vaccines13070721)
Supplement: Supplementary file 1 [file vaccines-13-00721-s001.zip › vaccines-3606214-supplementary.pdf]

Supplementary material

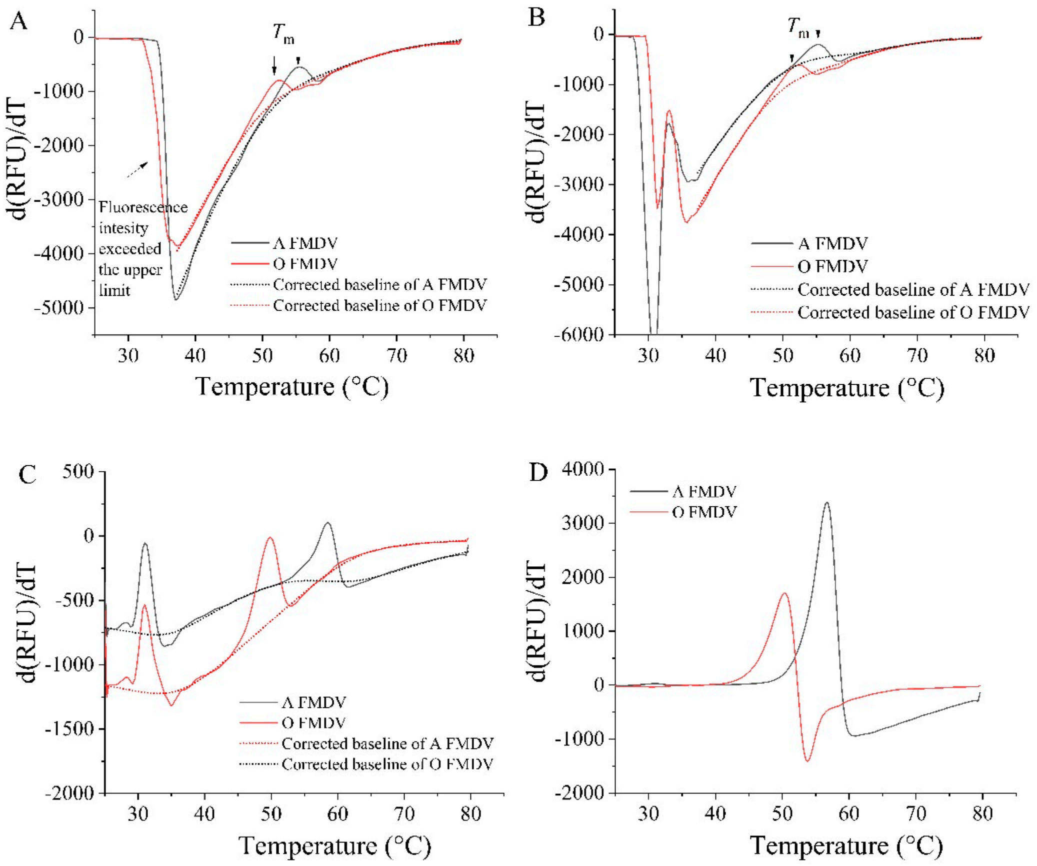

Figure S1. The first derivative plots of the DSF fluorescence curves of the FMDV from different processing steps. (A) Cell culture crude, (B) inactivated cell culture crude, (C) semi-purified FMDV, (D) purified FMDV. The large amount of residual host nucleic acids in cell culture crude was digested by benzonase to eliminate its strong disturbance of the FMDV signals. However, the baseline of DSF for impure FMDV will still be influenced. Therefore, the baselines of the first derivative plots for (A), (B), and (C) were corrected in asymmetric least squares smoothing mode and subtracted to obtain the maximum d(RFU/dT).
